# Exploring the Safety and Efficacy of Medical Termination of Pregnancy: A Comprehensive Review

**DOI:** 10.7759/cureus.46444

**Published:** 2023-10-03

**Authors:** Rajshree Leichombam, Dushyant Bawiskar

**Affiliations:** 1 Community Medicine, Jawaharlal Nehru Medical College, Datta Meghe Institute of Higher Education and Research, Wardha, IND; 2 Sports Medicine, Abhinav Bindra Sports Medicine and Research Institute, Bhubnaeshwar, IND

**Keywords:** 24 weeks, misoprostol, mifepristone, abortion, gestational age

## Abstract

Medical termination of pregnancy, also known as medication abortion, is a safe and effective method of terminating pregnancies in the early stages. It involves using medications, such as mifepristone and prostaglandin, to induce a miscarriage. The success rates of medical abortion vary depending on factors such as gestational age and the specific medications used. For pregnancies that are 49 days or less, the success rates range from 92% to 98%. The choice between misoprostol and gemeprost, both prostaglandins, does not significantly impact the outcomes. It is important to note that various factors, including study design, definitions of success, and prior experience with medical abortion may influence success rates. Strict criteria for success and limited familiarity with the procedure may result in lower reported success rates. Medical termination of pregnancy should be carried out under the guidance and supervision of healthcare professionals. It is crucial to consult a healthcare provider to receive accurate information, personalized guidance, and appropriate support throughout the process. Each situation is unique, and decisions regarding medical termination of pregnancy should be made in collaboration with a trusted healthcare provider.

## Introduction and background

The intentional termination of a pregnancy before the foetus can survive on its own outside the uterus is referred to as an abortion, often known as an induced abortion [1]. Due to the moral, ethical, theological, and socio-cultural ramifications of abortion, it is a complicated and divisive subject that provokes various ideas and feelings. There are several reasons why someone could decide to get an abortion. Unwanted pregnancies, contraceptive failure, financial or personal limitations, worries about maternal or foetal health, foetal abnormalities or genetic illnesses, or situations such as rape or incest are a few of the causes. The choice to undergo an abortion is very personal and may be affected by various circumstance-specific considerations. There are considerable regional and national differences in the accessibility and legality of abortion services [2].

Abortion is permitted and accessible in some locations, enabling people to seek safe, regulated treatments from medical experts. Some people may turn to risky and perhaps lethal procedures since abortion is restricted, only permitted under specific conditions, or even outright prohibited in other countries. To safeguard the health and well-being of those seeking abortions, it is crucial to make the provision of safe and legal abortion services a priority [3,4]. For the protection of reproductive rights and the ability of people to make knowledgeable choices about their bodies and futures, access to comprehensive reproductive healthcare, including family planning, contraceptive methods, and safe abortion services, is essential [5]. There are considerable regional and national differences in the accessibility and legality of abortion services. Ultimately, a person should decide whether to have an abortion after consulting with medical specialists and considering their circumstances, values, and beliefs [6]. While working towards comprehensive reproductive healthcare that serves the needs of every person, it is crucial to prioritize the health, safety, and reproductive rights of those seeking abortions [7].

## Review

Search methodology 

A comprehensive and detailed strategy was followed while researching the said topic: “medical termination of pregnancy and its consequences”. The results were chosen from research databases such as PubMed, Google Scholar, MEDLINE, Embase, etc. and thoroughly researched with keywords like “MTP”, “complications in abortions”, “surgical regimen”, and “medical regimen”. Articles in languages other than English were filtered out and not taken into consideration. The editor's note was also filtered out. Articles were included from 2000 to date. Following (Table 1) are some of the articles included in the review.

**Table 1:** List of studies included in the review

Author	Year	Type of Study	Conclusion
Zareba et al. [3]	2020	Research article	The major issue with the inability to terminate pregnancies in Poland has less to do with a specific person (medical staff) or the potential for using the conscience clause.
Lockshin et al. [8]	2020	Research article	Pregnant women having autoimmune disorders have some complications in terminating the pregnancy.
Micks et al. [9]	2012	Research article	It is safe and efficient to put macaques to sleep medically. As a starting procedure, the use of misoprostol 200 mcg buccalized and mifepristone 20 mg intramuscularly is advised.
Cameron et al. [10]	2022	Review	Abortion is a very safe operation, and complications are rare when it is carried out in a safe environment.

Abortions

Abortion, commonly referred to as medical termination of pregnancy (MTP), is a treatment used to stop a pregnancy before the foetus can live outside the uterus. It entails intentionally ending a pregnancy to avoid giving birth [8]. Making the decision to have an MTP is a difficult and intensely personal one that is frequently impacted by various variables, including one's own circumstances, health concerns, socioeconomic reasons, and personal views. There are a number of reasons a woman could decide to have her pregnancy medically terminated. Unwanted or unexpected births, health hazards to the mother or foetus, foetal abnormalities or genetic illnesses, and societal and personal circumstances like poverty or a lack of support are some of these causes [11,12]. Notably, laws governing MTP vary between various nations and areas, with some allowing complete access to abortion services. In contrast, others place tight restrictions on it or outright forbid it [1]. To safeguard women's reproductive rights, preserve their health and well-being, and lower the dangers associated with unsafe abortion practices, access to safe and legal MTP services is essential.

Depending on the gestational age of the pregnancy, numerous medical methods can be used to stop the pregnancy. Early-term abortions, usually performed during the first trimester, can be performed either medically (referred to as a medical abortion) or surgically (referred to as a suction or aspiration abortion). More invasive procedures, such as dilatation and evacuation (D&E) or induction abortion, may be necessary for later-term abortions. Regardless of the technique, thorough counseling, medical direction, and support from healthcare experts should ideally be involved in seeking a medical termination of pregnancy [13]. Prioritizing the security, welfare, and autonomy of women seeking abortion services is crucial, as is considering the procedure's ethical and legal implications. When contemplating or pursuing a medical termination of pregnancy, it's critical to consult with licensed healthcare experts and to abide by the rules and regulations of one's particular jurisdiction [14].

In one study, the impact of medical termination of pregnancy with respect to the status of previous pregnancies was studied among 403 women. The pregnancy period was considered to be ≤ 49 days. Among all subjects studied, 349 women followed the basic regimen of abortion and aborted the foetus. In recent years, both patients and physicians have embraced the use of mifepristone and misoprostol for medical termination [15]. As the use of medical termination of pregnancy rises, it is crucial to identify individuals at a high risk of failure. This study unequivocally shows that women with any prior pregnancy, especially term pregnancies, whether ended by caesarean section or spontaneous delivery, are more likely to need surgical intervention compared to primigravid women after administration of mifepristone or misoprostol for medical termination of pregnancy [16,17]. This finding is consistent with a number of previously published studies, even though some of those reports did not discover any connection between parity and the requirement for surgical intervention.

In a study by Micks et al., the facility for uterine evacuation in Western Galilee was available only at the obstetrics and gynecology department of their hospital, and so women in that region seldom traveled elsewhere for uterine evacuation [9]. However, they did not contact the patients to see if they had had surgical uterus evacuation at any other hospital, and this was a shortcoming of their study. Additionally, individuals who have this surgery pay in advance, and the cost of the operation covers the management of any difficulties and, if necessary, a surgical uterine evacuation. In addition, the hospital's Institutional Review Board prohibited calling patients due to privacy and confidentiality issues involving prior pregnancies [18,19].

Regimens for MTP

There are mainly two regimens available for the medical termination of pregnancy to be in effect. The first is the medical regimen, which is non-surgical, and the other is the surgical regimen. In the medical regimen, usually, medicines are given to terminate the pregnancy. If MTP must be performed non-surgically, physicians frequently suggest the medication mifepristone (Mifeprex) [20]. This medication can be administered orally or by injection. Antibiotics are also administered to prevent infection. The mifepristone blocks progesterone and the uterine lining is impacted by its absence. This decrease in progesterone stops the pregnancy from developing further. This procedure might take a few hours or days, resulting in contractions that expel the foetus. A medical assessment is also performed a week later to ensure the full termination of the pregnancy and rule out any problems [21-23].

Medical Regimen

Mifepristone, referred to by the brand names Mifeprex and RU-486, is a drug used for emergency contraception and medical abortion. It is a synthetic steroid substance that opposes progesterone, a hormone necessary to sustain pregnancy. The progesterone receptors are blocked by mifepristone, which causes the cervix to relax and the embryo to separate from the uterine wall.

Mifepristone is frequently used with the drug misoprostol, which aids in bringing on contractions and ending the pregnancy. For up to 10 weeks of gestation, mifepristone and misoprostol are thought to be an efficient and secure way to stop an early pregnancy [3]. The medicine is often given in accordance with a set regimen and under medical supervision. Within 24 to 48 hours of taking mifepristone, a second appointment with the doctor is planned for misoprostol administration [24]. It is crucial that people have access to the right medical treatment and support during this time because the process can include many hours of bleeding and cramping while the uterus empties. In some circumstances, mifepristone is used to treat specific medical diseases, including Cushing's syndrome, which is marked by high cortisol hormone levels. It has also been researched for possible applications in other medical fields, including cancer treatment and as an emergency contraceptive when used at greater dosages [25].

Abortion is largely linked to the socio-cultural aspects of human civilization. In most countries, it is highly regulated, and statutory laws are in place to regulate the procedure. Manual vacuum aspiration (MVA), electric vacuum aspiration (EVA), dilation and curettage (D&C), D&E, and induction abortion are some commonly used surgical methods to terminate a pregnancy [26]. An MVA is used up to 12 weeks into pregnancy, in which manual suction and a syringe are used to empty the material in the uterus. If an electric pump is used instead of a manual, then it is called an EVA. The D&C is also used in the first trimester. The third trimester is between 13 and 24 weeks, where surgical forceps and another instrument are used to evacuate the uterus. For the second trimester and beyond, the induction abortion method is used to terminate the pregnancy. This procedure requires close monitoring and health care infrastructure, as certain medications and complicated surgery can endanger the life of the affected individual [26-28].

Abortion's psychological effects on women might differ greatly from person to person. It is crucial to understand that each woman's experience with abortion is distinct and that personal factors, including values, circumstances, and support networks, may impact the psychological impacts. Following an abortion, some women could feel relieved or empowered, while others might go through a range of emotional reactions, such as sadness, guilt, grief, worry, or even a sense of loss. It is important to highlight that extensive study on the psychological repercussions of abortion has produced conflicting findings, and there is no agreement among scientists as to the scope or character of any long-term psychological impacts [29-32].

Surgical Regimen

Depending on the gestational age, regional laws, and preferences of the healthcare professional, the surgical procedure for medical termination of pregnancy, also known as an induced abortion, may differ. The MVA, EVA, and D&C are the three surgical regimens that are generally followed while attempting medical termination of pregnancy. The MVA is a surgical treatment frequently performed to end a pregnancy medically in the first trimester (up to 12 weeks gestation) [27].

A healthcare professional does the MVA by gently sucking the uterus's contents using an electric pump or handheld syringe. The technique normally takes 5 to 10 minutes to complete and is often carried out under local anesthesia or conscious sedation. In contrast to MVA, which uses a handheld syringe, EVA uses electric suction equipment. It is frequently used to end pregnancies medically in the first trimester (up to 12 weeks of gestation), and in some circumstances, it may even be used in the second trimester [28].

Impact on women

According to many well-designed studies, most women who have abortions do not suffer from substantial long-term psychological effects. Nevertheless, some women may feel momentary mental discomfort or manifest signs of sadness or anxiety after the surgery. There are a number of elements that could enhance adverse emotional reactions. Such as lack of social support, i.e., women are more susceptible to negative emotional reactions if they feel alone or don't have the support of their spouses, families, or friends; pre-existing mental health conditions; ambivalence or conflict about the choice resulting in conflicting emotions, moral or ethical dilemmas, or unsolved concerns; external pressures or stigma that impose guilt and shame; and ineffective coping mechanisms. It's critical to underscore that women who endure emotional anguish after an abortion must seek emotional care. Women with emotional difficulties should seek help from counselors, support groups, or mental health specialists trained in reproductive health or post-abortion care. These experts may offer tools, support, and direction in a nonjudgmental manner to assist women in processing their feelings and promoting recovery [33-35].

It's also important to note that many women report feeling relieved and resolved after having an abortion, especially when the choice fits with their values and circumstances. It's crucial to handle the issue of the psychological effects of abortion sensitively and with respect for individual experiences because it is a complicated and nuanced one.

Medical abortions, often known as pharmaceutical or non-surgical abortions, may include the use of a number of medicines. Different drugs may be prescribed depending on the nation, the medical facility, and the gestational age of the pregnancy. Mifepristone, usually referred to as RU-486 or the abortion pill, is an anti-progesterone drug that prevents the hormone progesterone from acting as a pregnancy-maintaining hormone. Typically, it is taken orally as tablets. Misoprostol is often administered after the medicine mifepristone in a hospital or clinic setting [36].

Misoprostol is a prostaglandin analogue that induces uterine contractions and aids in delivering the unborn child. It is often consumed for days to a few hours following mifepristone. Depending on the directions from the healthcare professional, misoprostol may be consumed orally, buccally (put between the gums and cheeks), or vaginally. Mifepristone and misoprostol together successfully end pregnancies when they are still in the early stages (up to 10 weeks of gestation) [27]. Although these drugs are often considered safe, they can have risks and adverse effects. For precise and individualized information on using these medications for abortions, it's vital to speak with a healthcare professional. This is because the procedures may change based on local laws and medical standards [36].

Prenatal screening techniques that are often used in China include chorionic villus sampling, amniocentesis, and ultrasonography. According to the degree of foetal anomalies, pregnancy outcomes in China are often categorized into three groups following multidisciplinary consultation: (1) The informed consent principle is followed, and parents are advised to have an induced abortion if the diagnosis is clear and certain to result in foetal death. (2) Doctors will notify parents of the perinatal prognosis for nonfatal foetal deformities. If parents want to end the pregnancy, they sign informed consent. (3) Doctors would encourage parents to visit a pediatrician for follow-up after the baby is delivered if they choose post-birth therapy. The psychological effects of receiving a foetal abnormality diagnosis for women have been studied in the past using qualitative research approaches. Women have described feeling unprepared, agonized by the delays, and shocked, and that the hardest part of the procedure was choosing a decision. It's typical to experience self-blame and anguish after making a termination choice. Additionally, women described intense physical and mental suffering before, during, and after the abortion [32].

The effectiveness of using mifepristone and prostaglandin in terminating pregnancies lasting 49 days or less ranges from 92% to 98%. The choice between misoprostol and gemeprost does not significantly impact the outcomes. However, it should be noted that a multicenter U.S. trial reported a 92% success rate, which might have been influenced by a stringent definition of success (requiring abortion within 15 days after mifepristone administration) and limited prior experience with medical abortion [34].

In cases where mifepristone is combined with oral misoprostol for pregnancies lasting 50 to 63 days, the success rate is often lower, ranging from 77% to 95%. On the other hand, combining mifepristone with gemeprost or vaginal misoprostol typically yields higher success rates, ranging from 94% to 97% [10].

The link between access to contraception and access to pregnancy termination

Aspects of reproductive health and family planning, such as access to contraception and access to abortion, are interconnected. Both of these are essential in granting individuals autonomy over their reproductive options and decisions. Unwanted pregnancies are avoided using contraception techniques, including birth control tablets, condoms, intrauterine devices (IUDs), etc. Individuals and couples may manage their families according to their tastes, financial stability, and life situations by having simple access to several contraceptive methods. As a result, fewer unintended pregnancies can lead to healthier pregnancies and better child-rearing circumstances. Pregnancy termination rates can be considerably reduced when reliable contraception is available. Unwanted pregnancies are one of the main reasons individuals choose to get an abortion. Societies may aim to lower the need for abortion services by ensuring that individuals have access to information about contraception.

## Conclusions

Medical termination of pregnancy, also known as medication abortion, is an effective and widely used method for terminating pregnancies in the early stages. The combination of mifepristone and prostaglandin, such as misoprostol or gemeprost, is commonly utilized for this purpose. For pregnancies of 49 days or less, the success rates of medical abortions with mifepristone and prostaglandin range from 92% to 98%. The choice between misoprostol and gemeprost does not significantly affect the outcomes. When mifepristone is combined with oral misoprostol for pregnancies lasting 50 to 63 days, the success rate is often slightly lower, ranging from 77% to 95%. However, combining mifepristone with gemeprost or vaginal misoprostol in these cases typically yields higher success rates, ranging from 94% to 97%. Every situation is unique, and medical decisions should be made in collaboration with a trusted healthcare provider.
